# The Role of Radiomics in Salivary Gland Imaging: A Systematic Review and Radiomics Quality Assessment

**DOI:** 10.3390/diagnostics12123002

**Published:** 2022-12-01

**Authors:** Giacomo Aringhieri, Salvatore Claudio Fanni, Maria Febi, Leonardo Colligiani, Dania Cioni, Emanuele Neri

**Affiliations:** Department of Translational Research, Academic Radiology, University of Pisa, 56124 Pisa, Italy

**Keywords:** radiomics, salivary glands, radiomics quality score, parotid, submandibular, sialadenitis, salivary tumor, xerostomia

## Abstract

**Background**: Radiomics of salivary gland imaging can support clinical decisions in different clinical scenarios, such as tumors, radiation-induced xerostomia and sialadenitis. This review aims to evaluate the methodological quality of radiomics studies on salivary gland imaging. **Material and Methods**: A systematic search was performed, and the methodological quality was evaluated using the radiomics quality score (RQS). Subgroup analyses according to the first author’s professional role (medical or not medical), journal type (radiological journal or other) and the year of publication (2021 or before) were performed. The correlation of RQS with the number of patients was calculated. **Results**: Twenty-three articles were included (mean RQS 11.34 ± 3.68). Most studies well-documented the imaging protocol (87%), while neither prospective validations nor cost-effectiveness analyses were performed. None of the included studies provided open-source data. A statistically significant difference in RQS according to the year of publication was found (*p* = 0.009), with papers published in 2021 having slightly higher RQSs than older ones. No differences according to journal type or the first author’s professional role were demonstrated. A moderate relationship between the overall RQS and the number of patients was found. **Conclusions**: Radiomics application in salivary gland imaging is increasing. Although its current clinical applicability can be affected by the somewhat inadequate quality of the papers, a significant improvement in radiomics methodologies has been demonstrated in the last year.

## 1. Introduction

In the past few years, the need for personalized and precision medicine has led to an increasing interest in radiomics, which is the transforming of digital images into mineable high-dimensional data through the extraction of hundreds of quantitative features [1]. The features extracted during the radiomics process may provide relevant information on cellular, metabolic and genomic aspects. Currently, these data cannot be provided by radiologists to referring physicians since these features are generally not visible to the naked eye and, thus, cannot be included in the radiological report.

Despite the enormous potential of radiomics and the convincing results recently published, its current use is limited to research, with few clinical applications. Indeed, the radiomics workflow consists of many steps, each one characterized by a myriad of factors, leading to variability that deeply affects study repeatability [2].

The need for scientific rigor led Lambin et al. to develop a standardized system of metrics called the radiomics quality score (RQS), which is a point-based system used to evaluate the validity and completeness of radiomics studies [3]. Radiomics has found several applications, ranging from diagnosis to prognosis, for oncological and other diseases of every organ in the human body, including the salivary glands. The major salivary glands comprise the three pairs of parotid, submandibular and sublingual glands, while the minor salivary glands comprise several hundred salivary glands located throughout the submucosa of the nasal cavity, oral cavity, and pharynx, as well as even the larynx, trachea, lungs, and middle ear cavity. Together, the major and minor salivary glands produce saliva, which has a fundamental role in the protection of the oral cavity mucous membrane and in the digestion process [4].

The major and minor salivary glands, as an organ system, have one of the greatest diversities of pathology, ranging from development abnormalities to post-traumatic changes. However, the three most important pathologies addressed by radiomics applications are benign and malignant tumors, inflammatory diseases and radiation therapy-induced xerostomia.

Different types of tumors can affect the salivary glands, with an estimated worldwide annual incidence of up to 2 per 100.000 individuals [5]. The most frequent localization of salivary gland tumors is the parotid, which is affected in 80% of cases by benign tumors, such as pleomorphic adenomas and Warthin tumors, while parotid malignancies are more rarely diagnosed [6].

Conversely, when considering the submandibular and sublingual glands, malignant tumors are more frequent, and among them, the most common are adenoid cystic carcinomas.

Regarding inflammatory diseases, sialadenitis usually presents with pain, swelling and xerostomia [7]. Sialadenitis recognizes a wide variety of causes, such as bacterial or viral infections; obstructive disease; and systemic disease, for example, Sjögren’s syndrome and IgG4-related disease [8,9,10]. Sjögren’s syndrome is an autoimmune disease affecting both the lacrimal and major salivary glands, and it occurs alone or might be associated with other connective tissue diseases. Another relevant cause of sialadenitis is related to radiotherapy of the oral cavity or pharyngeal tumors, which may lead to xerostomia. The parotid gland is more severely affected by radiotherapy compared to the other salivary glands [7].

Many imaging techniques are available for the salivary glands, including plain radiography, sialography, ultrasonography (US), computed tomography (CT), magnetic resonance imaging (MRI), radionuclide scintigraphy and positron emission tomography–computed tomography (PET-CT). No imaging technique can be considered strictly superior to another one, and the optimal method of investigation may change according to the clinical scenario [11].

This systematic review aims to analyze the current status of radiomics applications in salivary gland imaging and to assess the quality of the radiomics workflow by adopting the radiomics quality score.

## 2. Materials and Methods

### 2.1. Literature Search

Two reviewers independently performed a systematic literature search for potentially relevant articles about radiomics-based models in salivary gland imaging.

The examined electronic databases were PubMed, Scopus and Scholar. To achieve the highest search sensitivity, the following search terms were used to identify relevant articles: “parotid gland OR submandibular gland OR salivary gland AND radiomics”. The terms were chosen to include the imaging of all relevant disorders affecting the salivary glands and to explore the potential role of radiomics in their management, from diagnosis to the therapy assessment. For the Google Scholar electronic database, because of the excessive amount of data retrieved, only the first 800 results were screened due to the lack of relevance of further results.

After duplicate elimination, all the selected articles were initially screened by reviewing their titles and abstracts.

Two authors, S.C.F. and M.F., independently screened the titles of the identified papers. Another author (G.A.) independently screened the titles and the abstracts of the studies that passed the title screening; finally, the authors read the full text of the studies that successfully passed the title and abstract screening. Any disagreement was overcome by discussion to reach a mutual agreement. Filters were applied to include only original research published in English, considering articles published before 15 January 2022. No restrictions related to the country of publication, study design or outcomes were applied. The last search was run on 15 January 2022.

From each study, the following data were extracted: publication year, journal type (radiological journal or other), the number of patients, imaging modality, study design, the professional role of the first author (a medical doctor or not a medical doctor) and study aim.

### 2.2. Radiomics Quality Score

The methodological quality evaluation was carried out using the radiomics quality score (RQS) according to Lambin et al. [3]. RQS is a tool made up of 16 items, with different maximum scores in relation to each item’s importance. Domain 1 addresses protocol quality and the reporting of multiple segmentation or imaging at multiple time points.

Domain 2 considers the presence of feature reduction and the presence/absence and type of validation. Domain 3 concerns the presence of a demonstrated biological validation, a comparison to a gold standard and, finally, potential clinical utility.

Domain 4 addresses statistical analyses and the performance of the model.

Domain 5 is about the study design and the reporting of a cost-effectiveness analysis.

Finally, Domain 6 concerns evidence of open science and data. An overview of the RQS items, criteria and points is shown in Table 1.

Two reviewers (M.F. and L.C.) independently assigned the RQS absolute values to the selected studies. Then, the relative percentages were calculated. The summed total score ranges from −8 to 36, and the respective percentage scores are defined as 0% from −8 up to 0 and as 100% for 36. Conflicts between the two reviewers were resolved by consensus together with a third reviewer (S.C.F.).

### 2.3. Statistical Analysis

The overall RQS for the included studies was calculated by using the algebraic sum of each single RQS item, and, finally, the total RQS value of each study was reported as a percentage. The Shapiro–Wilk test was used to assess the normality of distribution for continuous variables. Subgroup analyses were performed using the Mann–Whitney U test to investigate whether the overall RQS varied significantly according to the professional role of the first author (medical or not medical), journal type (radiological journal or other) and the year of publication (2021 or before 2021). To measure the correlation of the overall RQS with the number of patients, the Spearman’s rank-order correlation was calculated. *p*-values less than 0.05 were considered to indicate statistical significance. All analyses were conducted using “IBM SPSS Statistics” (v25.0).

## 3. Results

**Figure 1:**
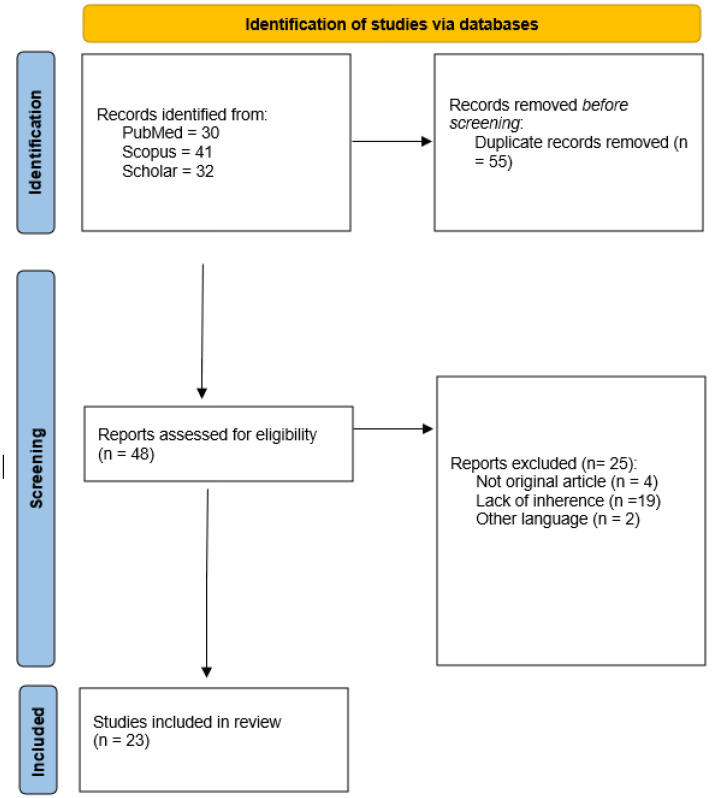
Study selection process flowchart according to the PRISMA Statement 2020 [12].

**Figure 2:**
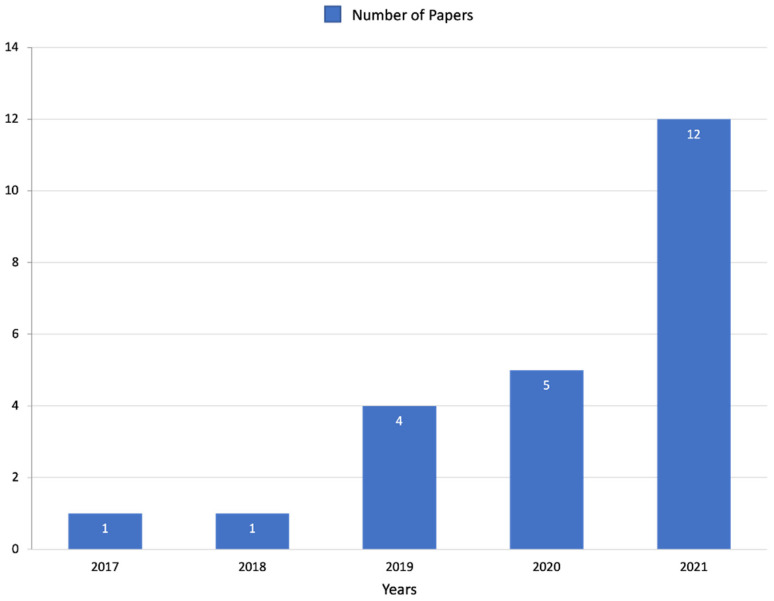
Distribution of reviewed papers from 2017 up to 2021.

A flowchart of the study selection process is reported in Figure 1. A total of 55 duplicates and 25 unrelated papers were removed, leading to the final inclusion of 23 articles in the review. The mean patient number was 158.52 ± 136.46 (range 18–626). Most of the articles (52%) were published in 2021, in radiological journals (65%) and with a medical doctor as the first author (82%). Five papers were published in 2020, four were published in 2019, one was published in 2018, and one was published in 2017 (Figure 2). None of the included articles were published before 2017. Regarding the study aims, most of the papers focused on a differential diagnosis (60%), followed by the assessment of radiation-therapy-related side effects (22%), diagnosis and staging (4%) and, finally, prognosis (4%). The most frequently addressed topic was oncology (70%), followed by the prediction of radiation-induced xerostomia (21%). Only two papers (8%) discussed radiomics applications in inflammatory diseases of the salivary glands. All the included papers were retrospective studies. Only one article (4%) employed a machine learning technique to reduce dimensionality and to select features, while slightly under half of the articles (43%) employed a machine learning technique for model building.

The characteristics of the included articles are summarized in Table 2.

Overall, the included studies achieved a mean RQS total of 11.34 ± 3.68 (range 3–16) corresponding to a percentage of 31.27 ± 10.40%. A detailed RQS assessment of all the included studies is shown in Table 3. Moreover, the mean of the total RQS and the corresponding percentage of the included articles categorized according to imaging technique used are reported in Table 4.

Most of the included articles well-documented the imaging protocol (87%), and in about half, multiple segmentations (43%) were performed. All the studies carried out a feature reduction. In 3 out of 23 studies (13%), validation is missing. A total of 14 out of 23 studies (61%) performed validation with an internal dataset, 5 performed validation with a single external institute (22%), and only 1 (4%) performed validation with three different external institutes.

Phantom studies, segmentations at multiple time points and prospective validations were not performed in any of the studies. Finally, no study performed a cost-effectiveness analysis or used open science data.

A statistically significant difference (*p* = 0.009) in the overall RQS between papers published in 2021 (11.9 ± 1.26) and those published before 2021 (10.45 ± 0.93) was found. No differences were demonstrated according to journal type or the first author’s professional role. A statistically significant moderate relationship (Spearman ρ = 0.43, *p* = 0.03) between the overall RQS and the number of patients was found.

## 4. Discussion

In salivary gland diseases, radiomics is able to provide several quantitative parameters potentially useful in supporting clinical decisions in different clinical scenarios, such as tumors, radiation-induced xerostomia and sialadenitis. Indeed, major and minor salivary gland tumors are rare entities, and clinical and imaging diagnoses based on US, CT or MRI can be very challenging, often needing further diagnostic invasive procedures, such as biopsies or fine-needle aspiration [36]. However, these invasive procedures do not always lead to a certain and definitive diagnosis due to inadequate bioptic samples, particularly in the deep lobe of the parotid gland [22,37]. Xerostomia is one of the most frequently described toxicities following radiation therapy, often combined with concurrent chemotherapy, of head and neck cancer, and it significantly affects long-term outcomes and the quality of life of patients. Unfortunately, however, our ability to characterize these side effects in individual patients and correlate them with radiotherapy dosimetry delivered to the salivary glands is currently limited [33]. Finally, for inflammatory diseases (e.g., submandibular sialadenitis and Sjögren syndrome), diagnosis or disease severity scoring can be challenging due to the presence of aspecific imaging findings and a significant inter-observer variability [18,38]. Due to all the above-mentioned challenges, interest in radiomics applied to salivary gland imaging has rapidly increased in recent years. Our results show that no paper had been published before 2017 and that more than half of the included papers were published in 2021 (52%).

Radiomics can be applied virtually to all imaging techniques, although it is preferentially used for more standardizable, cross-sectional examinations, such as CT and MRI, rather than US. In fact, in our review, only two studies carried out radiomics analyses on US images. The most frequently addressed clinical scenario was oncology (70%) compared with radiation-induced xerostomia (21%) and inflammatory pathology (9%).

This discrepancy reflects the need for radiologists to have more parameters and tools to differentiate tumors. Not surprisingly, regarding the study aim, most of the papers (60%) addressed differential diagnosis, the majority between benign and malignant tumors [15,16,22,23,25,27].

However, some of the included articles also specifically addressed the differential diagnosis between benign tumors, such as pleomorphic adenomas and Warthin tumors [16,20,22,24,26]. Indeed, although they are both benign tumors, the management and the therapy of these lesions are completely different; in pleomorphic adenomas, given the risk of transformation into carcinomas, parotidectomy is recommended [20].

Zheng et al. investigated the role of radiomics in the differentiation between benign lymphoepithelial lesions and mucosa-associated lymphoid tissue (MALT) lymphomas. The issue with these two lesions is that, due to the pathogenetic continuity existing between them, their imaging findings on CT and MRI mostly overlap [19].

Only one of the included papers studied the potential role of radiomics in PET-CT, which is also the only one with a prognostic purpose and the only one involving minor salivary gland carcinomas [29].

The five included papers that addressed the clinical scenario of xerostomia tried to identify radiomics features from MRI or CT scans extracted at baseline or in the weekly CT during treatment. These articles aimed to identify, in the very early stage or even before the beginning of radiation therapy, patients at risk of developing swallowing-related toxicities. Thus, radiomics may significantly influence the clinical decision-making, therapy planning and follow-up workflow for these patients [31,32,33,34,35].

According to our results, the possible applications of radiomics for salivary gland inflammatory diseases are still largely unexplored. The only two papers included in our review addressing this clinical scenario concern the development of radiomics-based scoring for Sjögren’s syndrome on salivary gland US and the differentiation between healthy normal submandibular glands and submandibular sialadenitis via a CT texture analysis, respectively [13,18].

To assess the methodological quality, all the 27 papers included in this systematic review were assessed using RQS [3]. The mean RQS was 11.34 ± 3.68, resulting in 31.27 ± 10.40%. This result is higher than the average results, with an RQS score of 18.87% reported in a systematic review of RQS applications published by the EuSoMII Radiomics Auditing Group [39]. This difference may be largely explained by the limits of the RQS tool itself. Indeed, most of the reviews using RQS to assess the quality of radiomics articles agree that RQS is a tool that lacks reproducibility and do not consider the different aims of different studies [40,41].

Our results on the first author’s professional role differ from those found by Ponsiglione et al., as we found no significant differences in RQS between medical and non-medical first authors [42]. Similarly, no differences considering the journal type were found.

However, no definitive conclusions can be drawn from these results due to the very limited number of papers reviewed, and further analyses are necessary as the number of papers increases.

Radiomics application in salivary glands is still far from everyday clinical practice; this may be explained by the limited number and the somewhat inadequate quality of the current publications on the topic based on RQS [40,41].

First, the papers presented a sample size that varied consistently depending on the specific topic. Second, all the papers used a retrospective design, with no prospective study strongly supporting its clinical application. Furthermore, the validation was not always adequate or even included, with only one paper using an external multicenter validation. Moreover, no paper provided public code or data, reducing the reproducibility based on transfer knowledge. Finally, the cost-effectiveness was not evaluated in any of the included articles.

Another key point to underline is the absence of feature robustness testing for multiple segmentations in more than half of the articles (57%). However, comparing our result to that of other reviews [43,44], where the authors stated that only very few articles included multiple segmentations, it can be considered a slight improvement.

Moreover, our data reported a positive trend for overall RQS as the number of enrolled patients increased. Considering that no RQS item assesses the numerosity of the study population, it can be hypothesized that, in studies with a higher numerosity, greater attention is paid to the study methodology. Finally, there was evidence of a significant improvement in the methodological quality of radiomics papers published in 2021 compared with those published in previous years. Undoubtedly, over the past few years, the increasing awareness in the scientific community has led to the higher methodological quality of papers about radiomics and its potential clinical applications.

## 5. Conclusions

In conclusion, this work demonstrates the increasing interest in radiomics application in salivary gland diseases, as in many medical fields, based on the clinical need for better diagnosis and management. Although its current clinical applicability can be affected by the overall somewhat inadequate quality of the reviewed papers, a significant improvement in radiomics methodologies in recently published papers has been demonstrated. Multicenter prospective studies with open-source code or datasets are needed to obtain higher reproducibility and to demonstrate the clinical utility of radiomics in salivary gland imaging.

## Figures and Tables

**Table 1:** Overview of RQS items, criteria and points.

Items	Criteria	Points
**Item1**	Image protocol quality—well-documented image protocols.	+1 (protocols well-documented) +1 (public protocol)
**Item2**	Multiple segmentations—possible actions are segmentation by different physicians/algorithms/software, perturbing segmentations by (random) noise, segmentation at different breathing cycles. Analyze feature robustness to segmentation variabilities.	+1
**Item3**	Phantom study on all scanners—detect inter-scanner differences and vendor-dependent features. Analyze feature robustness to these sources of variability.	+1
**Item4**	Imaging at multiple time points—collect individuals’ images at additional time points. Analyze feature robustness to temporal variabilities (e.g., organ movement, organ expansion/shrinkage).	+1
**Item5**	Feature reduction or adjustment for multiple testing—decreases the risk of overfitting. Overfitting is inevitable if the number of features exceeds the number of samples. Consider feature robustness when selecting features.	−3 (not implemented) +3 (implemented)
**Item6**	Multivariable analysis with non-radiomic features (e.g., EGFR mutation)—expected to provide a more holistic model. Permits correlating/inferencing between radiomics and non-radiomics features.	+1
**Item7**	Detect and discuss biological correlates—demonstration of phenotypic differences (possibly associated with underlying gene–protein expression patterns) deepens understanding of radiomics and biology.	+1
**Item8**	Cut-off analyses—determine risk groups by either the median or a previously published cut-off or report a continuous risk variable. Reduces the risk of reporting overly optimistic results.	+1
**Item9**	Discrimination statistics—report discrimination statistics (e.g., C-statistic, ROC curve, AUC) and their statistical significance (e.g., *p*-values, confidence intervals). One can also apply resampling methods (e.g., bootstrapping, cross-validation).	+1 (discrimination statistic with statistical significance)
**Item10**	Calibration statistics—report calibration statistics (e.g., calibration-in-the-large/slope, calibration plots) and their statistical significance (e.g., *p*-values, confidence intervals). One can also apply resampling methods (e.g., bootstrapping, cross-validation).	+1 (calibration statistics with statistical significance) +1 (and resampling method)
**Item11**	Prospective study registered in a trial database—provides the highest level of evidence supporting the clinical validity and usefulness of the radiomics biomarker.	+7 (prospective validation)
**Item12**	Validation—validation is performed without retraining and without adaptation of the cut-off value, provides crucial information with regard to credible clinical performance.	−5 (if validation is missing) +2 (validation with same) + 3 (with another institute) + 4 (with 2 datasets from two distinct institutes) +4 (validates a published signature) +5 (validation with dataset from ≥3 institutes)
**Item13**	Comparison to “gold standard”—assess the extent to which the model agrees with/is superior to the current “gold standard” method (e.g., TNM-staging for survival prediction). This comparison shows the added value of radiomics.	+2
**Item14**	Potential clinical utility—report on the current and potential application of the model in a clinical setting (e.g., decision curve analysis).	+2
**Item15**	Cost-effectiveness analysis—report on the cost-effectiveness of the clinical application (e.g., quality-adjusted life-years generated).	+1
**Item16**	Open science and data—make code and data publicly available. Open science facilitates knowledge transfer and reproducibility of the study.	+1 (open-source scans) +1 (open-source ROI) +1 (open-source code) +1 (open-source calculated features)
		Total points (36 = 100%)

Abbreviations: AUC: area under the curve, EGFR: epidermal growth factor receptor.

**Table 2:** Characteristics of included articles.

**Patient Number**		159 (mean, range 18–626)
**Journal type**	Radiological journal	15 (65%)
	Other	8 (35%)
Imaging modality *	US	2
	CT	10
	MRI	12
	[^18^F] FDG PET-CT	1
**Study aim**	Diagnosis and staging	3 (14%)
	Differential diagnosis	14 (60%)
	Assessment of therapy complications	5 (22%)
	Prognosis	1 (4%)
**Clinical scenario**	Oncology	16 (70%)
	Inflammatory disease	2 (9%)
	Radiation-induced xerostomia	5 (21%)
**Employment of Machine Learning technique**	To select features	1 (4%)
	To build predictive models	10 (43%)
**Nature of the study**	Retrospective	23 (100%)

* Two papers used both CT and MRI and are counted for each imaging modality.

**Table 3:** Radiomics quality scores of all included studies.

First Author (Years)	Item1	Item2	Item3	Item4	Item5	Item6	Item7	Item8	Item9	Item10	Item11	Item12	Item13	Item14	Item15	Item16	RQS (Total)	RQS (%)
Vukicevic 2020 (1) [13]	1	0	0	0	3	0	0	0	2	0	0	2	2	0	0	0	10	27.78
Vernuccio 2021 [14]	1	0	0	0	3	0	1	0	1	0	0	−5	2	2	0	0	5	13.89
Yuyun Xu 2021 [15]	1	1	0	0	3	1	1	0	1	1	0	3	2	2	0	0	16	44.44
Zhifen Xu 2021 [16]	0	1	0	0	3	1	0	0	0	0	0	5	2	0	0	0	12	33.33
Qunying Li 2021 [17]	0	0	0	0	3	1	1	0	1	1	0	2	2	2	0	0	13	36.11
Ito 2020 [18]	1	0	0	0	3	0	0	0	0	0	0	−5	2	2	0	0	3	8.33
Zheng 2021 (2) [19]	1	1	0	0	3	1	0	0	1	1	0	2	2	2	0	0	14	38.89
Yebo Liu 2021 (1) [20]	1	0	0	0	3	1	1	0	1	1	0	2	2	2	0	0	14	38.89
Wada 2019 [21]	1	1	0	0	3	0	1	0	2	0	0	2	2	0	0	0	12	33.33
Gabelloni 2020 [22]	1	0	0	0	3	0	1	0	1	0	0	2	2	2	0	0	12	33.33
Shao 2020 [23]	1	1	0	0	3	0	1	0	2	0	0	2	2	0	0	0	12	33.33
Zheng 2021 (1) [24]	1	1	0	0	3	1	1	0	1	1	0	3	2	2	0	0	16	44.44
Shao 2021 [25]	1	1	0	0	3	0	1	0	1	0	0	2	2	0	0	0	11	30.56
Song 2021 [26]	1	1	0	0	3	0	0	0	1	1	0	2	2	2	0	0	13	36.11
Yebo Liu 2021 (2) [27]	1	0	0	0	3	1	1	0	1	1	0	2	2	2	0	0	14	38.89
Zheng 2021 (3) [28]	1	1	0	0	3	1	0	0	1	1	0	3	2	2	0	0	15	41.67
Cheng 2020 [29]	1	0	0	0	3	0	1	0	1	0	0	2	2	0	0	0	10	27.78
Zhang 2021 [30]	1	0	0	0	3	0	1	0	1	0	0	−5	2	0	0	0	3	8.33
Pota 2017 [31]	1	0	0	0	3	1	0	0	2	0	0	2	2	0	0	0	11	30.56
Van Dijk 2019 [32]	0	0	0	1	3	1	0	0	1	0	0	2	2	0	0	0	10	27.78
Sheihk 2019 [33]	1	0	0	0	3	1	0	0	2	0	0	2	0	0	0	0	9	25.00
Van Dijk 2018 [34]	1	0	0	0	3	1	1	0	1	2	0	3	2	2	0	0	16	44.44
Yanxia Liu 2019 [35]	1	1	0	0	3	0	0	0	1	0	0	2	0	0	0	0	8	22.22

**Table 4:** Radiomics quality score of included studies categorized according to imaging modality.

Imaging Modality	RQS (Total)	RQS (%)
US (N = 2)	11.50 ± 2.12	31.94 ± 5.89
CT (N = 10)	10 ± 4.42	27.7 ± 12.28
MRI (N = 12)	12.41 ± 3.11	34.49 ± 8.65

## Data Availability

Not applicable.
